# Markov Model-Based Method to Analyse Time-Varying Networks in EEG Task-Related Data

**DOI:** 10.3389/fncom.2018.00076

**Published:** 2018-09-21

**Authors:** Nitin J. Williams, Ian Daly, Slawomir J. Nasuto

**Affiliations:** ^1^Neuroscience Center, Helsinki Institute of Life Science, University of Helsinki, Helsinki, Finland; ^2^Brain-Computer Interfaces and Neural Engineering Laboratory, School of Computer Science and Electronic Engineering, University of Essex, Colchester, United Kingdom; ^3^Biomedical Sciences and Biomedical Engineering Division, School of Biological Sciences, University of Reading, Reading, United Kingdom

**Keywords:** EEG/MEG dynamic connectivity, EEG/MEG time-varying networks, sparse-MVAR modeling, markov modeling, granger causality, effective connectivity

## Abstract

The dynamic nature of functional brain networks is being increasingly recognized in cognitive neuroscience, and methods to analyse such time-varying networks in EEG/MEG data are required. In this work, we propose a pipeline to characterize time-varying networks in single-subject EEG task-related data and further, evaluate its validity on both simulated and experimental datasets. Pre-processing is done to remove channel-wise and trial-wise differences in activity. Functional networks are estimated from short non-overlapping time windows within each “trial,” using a sparse-MVAR (Multi-Variate Auto-Regressive) model. Functional “states” are then identified by partitioning the entire space of functional networks into a small number of groups/symbols via *k*-means clustering.The multi-trial sequence of symbols is then described by a Markov Model (MM). We show validity of this pipeline on realistic electrode-level simulated EEG data, by demonstrating its ability to discriminate “trials” from two experimental conditions in a range of scenarios. We then apply it to experimental data from two individuals using a Brain-Computer Interface (BCI) via a P300 oddball task. Using just the Markov Model parameters, we obtain statistically significant discrimination between target and non-target trials. The functional networks characterizing each ‘state’ were also highly similar between the two individuals. This work marks the first application of the Markov Model framework to infer time-varying networks from EEG/MEG data. Due to the pre-processing, results from the pipeline are orthogonal to those from conventional ERP averaging or a typical EEG microstate analysis. The results provide powerful proof-of-concept for a Markov model-based approach to analyzing the data, paving the way for its use to track rapid changes in interaction patterns as a task is being performed. MATLAB code for the entire pipeline has been made available.

## 1. Introduction

In the last two decades, there has been a gradual change in the dominant paradigm for understanding the neural basis of cognition. In particular, the view that cognitive function is subserved by individual brain regions has been supplanted by recognizing the importance of functional integration between brain regions in large-scale brain networks (Bressler and Menon, 2010; Meehan and Bressler, 2012). Although this idea can be traced back to (Pavlov, 1949), Luria (1966), and (Wernicke, 1970) its emergence as a paradigm has been facilitated by the availability of non-invasive neuroimaging methodologies (*f* MRI, EEG, MEG), and gained acceptance through work of Mesulam (1990), Bressler (1995), Horwitz et al. (2000), McIntosh (2000), Sporns et al. (2004), and Bressler and Tognoli (2006) among others.

Traditionally, networks active during task or at rest have been considered static with respect to time and techniques to measure network interactions have largely reflected this assumption. However, conceptual frameworks proposed by for example Rabinovich et al. (2008) and Tognoli and Kelso (2004) are suggestive of dynamic changes in network structure. Under this view of brain function, both at rest and while performing a task, the brain is considered to alternate between *dwelling* in certain states and *rapid transitions* between these states. Each state is assumed to be represented by a particular functional network pattern. This view has been corroborated by biophysical network models emulating population-level brain activity, for example work by Deco and Jirsa (2012) and Hansen et al. (2015). The time-varying nature of functional networks has also been confirmed in empirical work, in both resting-state (Chang and Glover, 2010) and task-based experimental data (Sakoglu et al., 2010).

EEG/MEG, due to their fine temporal resolution and direct access to electrophysiological activity, are well suited to track these changes in functional networks with time. However, the analysis methods to be able to do this are still being developed. The first methods to capture the time-varying nature of cognitive neuro-dynamics were proposed in Lehmann (1971, 1972), and these methods were used to identify four classes of EEG “microstates” from resting-state EEG (Koenig et al., 2002). This was done by applying *k*-means cluster analysis to the set of vectors defining instantaneous scalp field potential distribution. The mean duration of each microstate was between 80 and 100 *ms*. (Allefeld et al., 2009) adopted a similar approach to describing time-varying nature of dynamics reflected in EEG activity. The instantaneous state reflected by the EEG activity was indicated by the global amplitude after locally matching ellipsoids to the multivariate EEG data. Two states were identified, corresponding approximately to the periods of normal and epileptic EEG activity.

While these methods do describe the time-varying nature of dynamics revealed by EEG, the scalp field patterns described are only indirectly related to the underlying functional networks of interest. Some recently proposed methods (Baker et al., 2014; Hirayama and Ogawa, 2014) also have a similar limitation. For example, the methods proposed in Baker et al. (2014) and Hirayama and Ogawa (2014) characterized states as realizations, respectively, of a multi-variate Gaussian process and Student's *t* distribution. These descriptions are also only indirectly related to the underlying functional network. Some other recent methods however (Ito et al., 2007; Daly et al., 2012; Vidaurre et al., 2016), do use characterisations which are likely to reflect the functional network. Daly et al. (2012) use complex network measures, i.e., mean clustering coefficient, to describe patterns of phase-locking at the level of EEG electrodes. These are used as observables to a Hidden Markov Model (HMM), which uses these observables to identify hidden states and probabilities of transitions between states. Similarly, Vidaurre et al. (2016)) use an MVAR (multi-variate auto-regressive) model to describe time-delayed dependencies of MEG source-level activity. The MVAR parameters are also used as observables to an HMM.

In parallel with the above methods which identify putative functional states from EEG/MEG activity, some other methods have adopted a different approach of simply determining functional connectivity (statistical dependencies) or effective connectivity (causal dependencies) patterns at successive time points (Ding et al., 2000; Valencia et al., 2008; Wilke et al., 2008; Sommerlade et al., 2009; Hu et al., 2012). For example, Ding et al. (2000) demonstrated how MVAR models could be estimated on short time segments by combining data from multiple trials - the adaptive MVAR approach. By fitting MVAR models to successive windows of 50 *ms*, they revealed rapidly changing dynamics during different stages of a GO/NO-GO task performed by macaques on an intracranial EEG study.

While this second group of methods (e.g., Ding et al., 2000; Valencia et al., 2008) use advanced estimation techniques to infer connectivity patterns on a sample-by-sample basis, a limitation is that they do not group these connectivity patterns into states and model transitions between states. For example, an elegant formulation of the adaptive MVAR approach within a state-space modeling framework was proposed by Wilke et al. (2008), using a Kalman-filter algorithm to estimate the time-varying coefficient matrices. The method was validated using simulated data as well as epileptiform EEG data, in which results from the method were consistent with clinical assessments performed by neurologists. Sommerlade et al. (2009) extended the adaptive MVAR approach further, by including two state spaces - one for the time-varying auto-regressive coefficients and one for the hidden variable itself. Hu et al. (2012) used a Kalman-smoothing algorithm rather than a Kalman-filter algorithm to estimate the time-varying auto-regressive coefficients, to avoid the estimation bias due to tracking lag from Kalman-filters. The method also produced plausible results when applied to experimental data from an SEP (somato-sensory evoked potential) paradigm. Recently, Brookes et al. (2014) applied windowed Canonical Correlation Analysis (CCA) to produce time courses of connectivity from MEG source-reconstructed time series and O'Neill et al. (2016) applied ICA to identify groups of connections which varied in a similar manner across time.

There is thus a need for a method which *both* identifies functional states (each represented by a unique functional network) and describes the transitions between these states, in a task-related EEG/MEG context. In this paper, we propose an analysis pipeline to achieve this at the single-subject level. Pre-processing is first done to remove channel-wise and trial-wise differences in activity. We then use an extension of MVAR modeling which assumes sparse networks (Valdés-Sosa et al., 2005), to define functional networks from short, non-overlapping time segments. *k*-means clustering is then performed to partition the entire set of networks into a small number of groups or symbols. Markov Modeling is subsequently employed to model this multi-trial sequence of symbols. This is the first time, to our knowledge, that the Markov Modeling approach has been applied to analyse time-varying networks from EEG/MEG data. Notably, results from the pipeline are orthogonal to results from conventional ERP averaging or a typical EEG microstate analysis.

We validate the pipeline on realistic simulated EEG data by demonstrating its ability to discriminate “trials” from two experimental conditions, in a range of scenarios. We further validate its use by establishing that it can discriminate “trials” from two experimental conditions, when applied to experimental EEG data from a BCI P300 oddball task. The next section (section Methods) describes the method in more detail as well as the investigations performed on simulated and experimental data. In section Results, we present results from simulations as well as results of applying the method to experimental data. In section Discussion, we discuss merits and limitations of the method, compare it to other methods which analyse time-varying connectivity, and propose how the method could be developed further.

## 2. Methods

**Figure 1:**
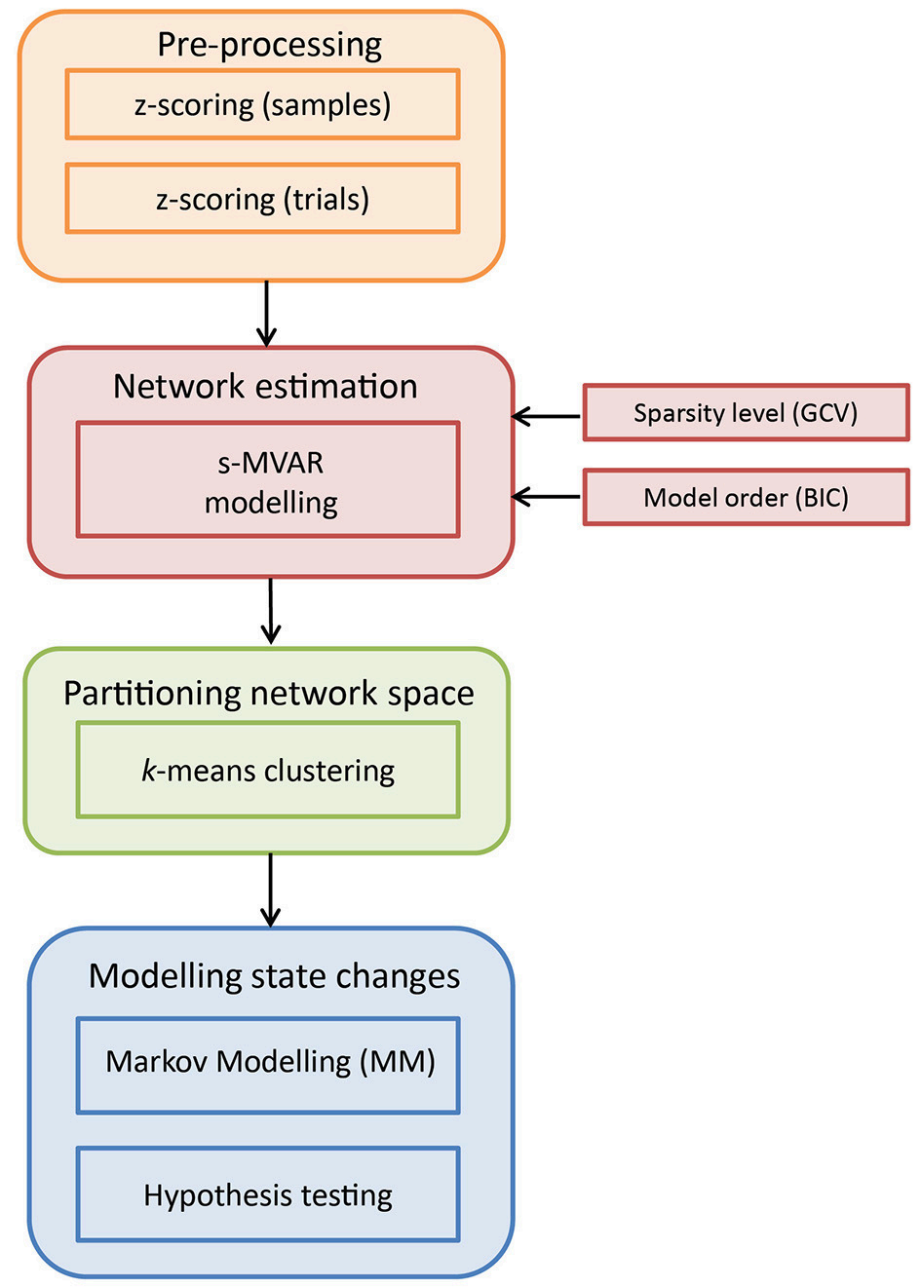
Schematic illustration of the analysis pipeline to characterize time-varying network dynamics. The input dataset is of dimensions *m* × *n* × *d*, where *m* is the number of channels, *n* is the number of samples and *d* is the number of trials across conditions. Pre-processing is performed by first *z*-scoring across samples and then, *z*-scoring across trials. Next, s-MVAR is employed to infer functional networks from short, non-overlapping windows. To identify putative functional states, the high-dimensional network space is partitioned using *k*-means clustering and each functional network is assigned a symbol or cluster number. For each condition, this multi-trial sequence of symbols is then modeled using a Markov Model (MM) and the estimated Markov model parameters from the two experimental conditions are then compared using non-parametric hypothesis testing.

The entire analysis pipeline is illustrated in Figure 1. The input dataset **Y** for a single participant can be considered to have dimensions *m* × *n* × *d*, containing multi-channel ERP data from multiple trials across experimental conditions. Here, *m* is the number of channels, *n* is the number of samples per trial, and *d* is the number of single-trials.

### 2.1. Pre-processing

As presented in Figure 1, the dataset is first pre-processed. Specifically, values representing activity on each channel were *z*-scored (subtracted by mean and divided by standard deviation) for each single-trial separately. This was done to make values comparable across trials. Then, at each sample, values were *z*-scored across trials (subtracted by mean across trials for each channel and divided by standard deviation across trials for each channel), for each condition separately. This was done to remove first-order and second-order sources of non-stationarity from the dataset, represented by the mean and standard deviation, respectively. Notably, subtracting the mean across trials from each channel amounts to removing the ERP (event-related potential) for that experimental condition, from the dataset. The above two pre-processing steps were recommended in Ding et al. (2000) for cases in which non-stationary network dynamics are being investigated.

### 2.2. Network estimation

Multi-Variate Auto-Regressive (MVAR) modeling is an established technique to characterize multi-channel EEG data (Ding et al., 2000). Both MVAR and measures derived from it such as Partial Directed Coherence (PDC) and Directed Transfer Function (DTF) have been interpreted in terms of information flow between channels. Mathematically, an MVAR model can be described as:

(1) $$\begin{array}{r} y_{i}\left(t\right)=\overset{m}{\underset{j=1}{\sum }}\overset{p}{\underset{k=1}{\sum }}a_{i,j}\left(k\right)y_{j}\left(t-k\right)+u_{i}\left(t\right)\;i=1,\ldots ,m \end{array}$$

where *y*_*i*_(*t*) is the current value at channel *i*, modeled as the linear-weighted combination of past values of all channels including itself, summed with white Gaussian noise *u*_*i*_(*t*). The weights are specified by *a*_*i, j*_(*k*) and the model order is *p*, indicating the number of preceding samples from each channel included in predicting the current value at a given channel. The functional network can then be calculated from the MVAR coefficients themselves, or from derived measures like PDC and DTF. A useful feature of the MVAR framework as well as the sparse-MVAR extension described below, is that functional networks inferred from the model parameters are directed in nature. Also, because of the multi-variate nature of the fit, the connections correspond to direct connections rather than mediating/indirect connections - assuming that all sources of interest are included in the modeling.

#### 2.2.1. s-MVAR modeling

For a given number of channels *m* and a model order *p*, the number of parameters to be estimated are *m* × *m* × *p* and the minimum number of samples required to estimate the model increases with the number of model parameters. In a task-related context, the MVAR model is generally estimated across trials, from short overlapping time-windows (Ding et al., 2000). However, the relationship between number of parameters in the MVAR model and the minimum number of samples required to estimate the model, limits the ability to track changes in functional networks with high temporal resolution. To infer networks from a smaller number of samples, Valdés-Sosa et al. (2005) proposed sparse-MVAR modeling, using penalized regression techniques under the assumption of network sparsity. We exploit this characteristic of s-MVAR to estimate functional networks from short non-overlapping windows, thus allowing for the investigation of fast changes in functional networks.

The s-MVAR model can be understood by expressing the original MVAR model as a multivariate regression,

(2) $$\begin{array}{r} \text{Y}=\text{XA}+\text{U}\;{\text{U}}_{i}\sim N\left(0,\Sigma \right), \end{array}$$

where **Y** is a *n* × *m* matrix (*n* being the number of samples), **A** is a *q* × *m* (where *q* is the product of *m* and *p*), **X** is a *n* × *q* matrix and contains time-lagged versions of **Y** up to the specified model order, and **U** is a *n* × *m* matrix of residuals. Σ is the covariance matrix of the residuals. Since estimating regression coefficients does not depend on the covariance matrix of residuals, each column of the MVAR coefficient matrix **A** can be estimated separately. This can be expressed as:

(3) $$\begin{array}{r} \hat{\alpha }=\underset{\alpha }{\arg\min}||\text{Y}-\text{X}\alpha |{|}^{2} \end{array}$$

where α are MVAR coefficients for a single column of **A**. When including a penalty function, specifically the L1 norm, the estimation becomes:

(4) $$\begin{array}{r} \hat{\alpha }=\underset{\alpha }{\arg\min}||\text{Y}-\text{X}\alpha |{|}^{2}+\lambda^{2}\overset{d}{\underset{j=1}{\sum }}|\alpha_{j}| \end{array}$$

In general, the L1 norm constrains the sum of absolute values of the regression coefficients to be low, thereby shrinking the values of the low coefficients toward zero. Therefore, for sparse functional networks, s-MVAR modeling can lower the number of samples required to estimate a functional network with a given level of accuracy. To determine the set of values which minimize the above function, we used a numerical optimization technique called the Majorization-Minorization algorithm (Hun and Lange, 2004), which is a generalization of the Expectation-Maximization (EM) method. This approach operates by constructing a simple surrogate function that minorizes (or majorizes) the original objective function, i.e., when the surrogate function is iteratively optimized, the objective function is driven up or down as needed. It reduces the complexity of the optimization problem, in particular by avoiding large matrix inversions, linearizing the optimization problem and turning a nondifferentiable problem into a smooth problem. Hence, it is computationally simple while also maintaining a reasonable (linear) rate of convergence. Please refer to Hun and Lange (2004)) for a more detailed account of the technique. The technique has been employed to infer functional networks from *f* MRI data (Valdés-Sosa et al., 2005), and we obtained a MATLAB implementation of the technique from the journal website of that paper.

To select the sparsity level λ, a Generalized Cross Validation (GCV) criterion was used. Specifically, models were estimated for a range of λ values and for each of these models, the GCV was estimated by

$$\begin{array}{l} GCV=\frac{RSS}{{\left(L-df\right)}^{2}} \end{array}$$

where *RSS* is the residual sum of squares from the multivariate regression, *L* is the number of independent observations and *df* is the estimated degrees of freedom in the multivariate regression (Hun and Lange, 2004). The λ value corresponding to the lowest GCV value was selected. *L* was set to the difference of the number of samples and the assumed model order.

The model order was selected as the order corresponding to the lowest Bayesian Information Criterion (BIC) value, for a range of model orders. BIC was calculated as

$$\begin{array}{l} BIC=log\left(\sigma \right)+\frac{\left(log\left(L\right)-1\right)df}{L} \end{array}$$

where σ was the error variance of the estimated model.

#### 2.2.2. Application of s-MVAR

After pre-processing, the data were split into non-overlapping windows of 50 ms or 12 samples (40 ms or 40 samples for simulations), so the number of windows for a 1 *s* trial was 20 (25 for simulations). The width of the windows was chosen to be half the assumed duration of functional states (80–100 *ms*), as reported in the EEG microstate literature (Koenig et al., 2002). This would allow to track even rapid changes in these states. The functional network for each window was derived from parameters of the s-MVAR model fitted to data in that window. In practice, the short windows contained too few samples to estimate the s-MVAR model reliably, so we combined data from a few successive trials to estimate the functional network, rather than estimating them for each single-trial separately. We did this by concatenating data from multiple trials, after removing mean across samples from each individual trial. Data from 10 trials (3 trials for simulations) were concatenated, to be able to estimate the s-MVAR model from 120 samples. This procedure assumed that the s-MVAR process was stationary across the concatenated trials. Please see Figure S1 for details of our investigations on performing multi-trial estimation of a s-MVAR model. The λ values were calculated for a randomly selected 10% of windows (1% for simulations) across experimental conditions, the average of these values was taken as the sparsity level. Model order was determined on another sample of randomly picked 10% of the windows, exploring orders between 1 and 6. Given the short window width, 6 was considered upper bound of model order. With the chosen λ value and model order, s-MVAR coefficients were estimated for each window, and organized into an *m* × *m* × *p* matrix. The absolute value of s-MVAR coefficients was taken and their mean computed along the dimension of model order, to furnish a characterization of the functional network. After the s-MVAR stage, each window is represented by an *m* × *m* matrix of functional connection strengths.

### 2.3. Partitioning space of functional networks

The entire set of functional networks constitutes a set of square matrices, considered as a set of points in high-dimensional space. To identify putative functional states (before modeling their transitions using a Markov model), we used *k*-means clustering as a way of coarse-graining the high-dimensional space and labeling each functional network by the region it occupies in this space.

#### 2.3.1. *k*-means clustering

*k*-means clustering is an iterative refinement technique formalized in Hartigan and Wong (1979), which partitions a dataset into *k* clusters. It does this by finding the set of *k* cluster centroids which minimizes the sum, over all clusters, of the within-cluster sums of point-to-centroid distances, where “point” is a functional network representation in our case. Typically, the cluster centroids are initialized by randomly selecting *k* points from the dataset and then, an *assignment* step and *update* step are performed iteratively until convergence or a maximum number of iterations (please see Hartigan and Wong, 1979 for details).

#### 2.3.2. Application of *k*-means clustering

We performed *k*-means clustering on the set of vectorized functional network representations. The vectors characterizing each of the functional networks were collected into a matrix. In this matrix, the number of rows was equal to the number of functional networks, i.e., the product of the number of experimental conditions, the number of trials (after combining) for each condition and the number of windows per trial. The number of columns was the number of elements in the functional network representation, after removing diagonal elements. The cluster centroids for the *k*-means algorithm were initialized by randomly choosing *k* rows from the dataset. Clustering was performed with 1,000 such initializations and the solution corresponding to the minimum value of the cost function (sum, over all clusters, of the within-cluster sums of point-to-centroid distances), was chosen. The distance measure used was 1-*r*, where *r* is the sample correlation between the given point and the cluster centroid. We chose correlation as the distance measure to reflect our assumption that functional states are distinguished by the *pattern* of the corresponding functional networks. While Euclidean distance is another valid option for a distance measure, we did not choose it because it confounds *strength* and *pattern* of connections when comparing functional networks. Since determining the number of clusters in a data-driven manner is prone to error, we explored results for *k* set from 2 to 8 (intervals of 1), the chosen range reflecting our assumption of a small number of functional states. The maximum number of iterations was set to 100. For the update step, both *batch update* and *online update* were performed for each iteration, i.e., cluster centroids were re-calculated on the basis of learning from from both the whole dataset and individual rows respectively.

### 2.4. Modeling state changes

After clustering, each functional network representation can be replaced by the respective cluster number, indicating the region it occupies in high-dimensional space. Hence, the responses for each experimental condition can be presented as a multi-trial sequence of symbols, where the number of rows is the number of trials (after combining) and the number of columns is the number of symbols per trial. For each experimental condition, this matrix can be characterized by a Markov Model.

#### 2.4.1. Markov modeling (MM)

Markov models (Rabiner, 1989) are a type of stochastic signal model which assume the *Markov property* i.e., that the next state of the system depends only on the present state and not on those preceding it. Thus, to identify a given Markov model, one needs to determine the distribution of probabilities over the initial state *P*(*S*_1_) and the *Q* × *Q* (where *Q* is the number of states) matrix of probabilities of going from each state to all states including itself.

The initial state probabilities are

$$\begin{array}{l} \pi_{i}=P\left(q_{1}=S_{i}\right)\;i=1,\ldots ,Q \end{array}$$

and the state transition probabilities are

$$\begin{array}{l} \alpha_{i,j}=P\left(q_{t}=j|q_{t-1}=i\right)\;i,j=1,\ldots ,Q \end{array}$$

#### 2.4.2. Application of markov modeling (MM)

The parameters of the Markov model were estimated using the Baum-Welch Expectation Maximization (EM) algorithm, implemented in the HMM (Hidden Markov Model) MATLAB toolbox developed by Kevin Murphy ^1^ (by fixing emission matrix to identity matrix). The vector of starting probabilities was initialized with values between 0 and 1, summing to 1. The matrix of transition probabilities was initialized as a randomly specified stochastic matrix, i.e., with elements of each row summing to 1. Furthermore, the number of states *Q* was set to be the number of clusters *k* in the dataset. The maximum number of iterations of the Baum-Welch algorithm was set to 100, and the convergence threshold was set to 1*e* − 04.

##### 2.4.2.1. Hypothesis testing

To determine the ability of the pipeline to discriminate between trials from two different experimental conditions, we used the performance measure of 'model distance', i.e., the distance between the estimated Markov models of the two experimental conditions. Model distance was calculated according to the definition in Rabiner (1989) as a measure of how well model *M*1 matches state sequences from model *M*2, compared to how well it matches state sequences from itself. Mathematically, this is defined as

$$\begin{array}{l} D\left(M1,M2\right)=\frac{1}{T}\left[logP\left(S^{\left(2\right)}|M1\right)-logP\left(S^{\left(2\right)}|M2\right)\right] \end{array}$$

where *T* is the number of symbols in a trial, and *S*^*c*^ is a sequence of states from model *c*. Since this measure of model distance is asymmetric, the average of *D*(*M*1, *M*2) and *D*(*M*2, *M*1) is typically taken. To ensure that log-likelihood is a finite value, transition probabilities of zero were replaced by an infinitesimally small value (2.2204*e*-16, i.e., *eps* in MATLAB) before corresponding log-likelihood was calculated.

To determine if the estimated model distance was statistically significant, we compared it to a corresponding null distribution. In order to make minimal assumptions about the null distribution, we performed hypothesis testing via non-parametric randomization tests. Specifically, we generated an empirical null distribution of “model distance” by applying the above procedure on 1,000 sets of random mixed (without replacement) trials from the two conditions. By comparing obtained 'model distance' with the lower tail of this null distribution, an approximate *p*-value was obtained.

We have made MATLAB code for the entire pipeline available for download ^2^.

### 2.5. Simulations

#### 2.5.1. Simulation 1 - MVAR and s-MVAR comparison

We first performed simulations to compare accuracies of estimating the MVAR coefficients with a conventional MVAR approach and a sparse-MVAR approach. The parameters of the simulations which were held constant were the number of sources (32), the model order (1) and the range of MVAR coefficient values (0.1 ≤ *x* ≤ 0.2). One of the parameters which was varied was the number of samples of the MVAR process, as a ratio of the number of parameters to be estimated (which was 32^2^ = 1024). The levels were 0.05, 0.1, 0.2, and 0.5. The number of non-zero MVAR coefficients was also varied, as a ratio of the number of parameters to be estimated. The levels were 0.05, 0.1, and 0.2 - these corresponded to sparse networks in which 5 , 10, and 20% of connections were present respectively. Finally, signal-to-noise (SNR), as calculated by the ratio of standard deviation of signal to standard deviation of white Gaussian noise, was also varied. The levels were 0.1, 1, 10 and ∞.

For each three-way combination of the factor levels, 40 replications were generated. In each replication, an MVAR process was generated with the given number of samples, network density and SNR, and estimated using both s-MVAR and MVAR approaches. The sparsity level parameter at each level of network density was set by estimating it from an independently generated dataset, using the GCV index previously described. This independent dataset had SNR=∞ and the number of samples was 10 times the number of parameters to be estimated.

The accuracy of estimation was quantified by the Root Mean Square Error (RMSE) between the actual MVAR coefficient matrix and the estimated MVAR coefficient matrix, lower values indicate more accurate estimation. Once this was done for all replications, for each three-way combination of factor levels, the s-MVAR, and MVAR performances were compared using a paired, one-tailed sign test, for each of the factors (number of time points, network density and SNR). For each of the factors, this tested the hypothesis that the median of the difference between the two samples was zero. The sign test was used instead of the *t*-test because the data were found to be non-Gaussian (using Lilliefors test). Since accurate network estimation (low RMSE) implies that not just the connection strengths but *also* the *pattern* of the functional network are well approximated, results from this test indicated which of the s-MVAR or MVAR modeling frameworks was better suited to our purpose of identifying functional states based on functional network patterns.

#### 2.5.2. Simulation 2 - pipeline validation

We generated simulated EEG datasets of two experimental conditions, each condition having different time-varying network structure. For this, we assumed three functional states per condition, where each state is represented by a different pattern of functional connections. To generate the sequence of states for a given experimental condition, we assumed a Markov process.

The transition probabilities for Condition 1 were set to:

(5) $$\begin{array}{r} {}[\begin{array}{ccc} 0.8 & 0.1 & 0.1\\ 0.05 & 0.8 & 0.15\\ 0.05 & 0.05 & 0.9 \end{array}] \end{array}$$

The transition probabilities for Condition 2 were set to:

(6) $$\begin{array}{r} \left[\begin{array}{ccc} 0.7 & 0.2 & 0.1\\ 0.1 & 0.8 & 0.1\\ 0.2 & 0.05 & 0.75 \end{array}\right] \end{array}$$

The high transition probabilities on the diagonal reflected our assumption that given the fine temporal scale (narrow window width) at which we track network changes, that the brain is likely is dwell in the same state at successive time points. A total of 300 trials were assumed for each experimental condition. The same sequence of states across trials was used for a given condition, as would be expected in a task-related EEG experiment. The number of states per trial was set to 25, thus the matrix specifying the sequence of states across trials had dimensions of 300 × 25 for each experimental condition.

As mentioned, each state was represented by a unique functional network pattern. The number of sources in the functional network was *M*, where *M* had three levels: 8, 16 or 32. Within the *M* × *M* connectivity matrix of possible connections for each state, 10 % randomly chosen connections were assigned strengths between 0.1 and 0.5, all others were set to 0. Hence, each of the 6 states (2 experimental conditions, 3 states each) had a different connectivity pattern.

Then, for every occurrence of a state in the 300 × 25 matrix specifying the sequence of states (for each condition), an MVAR process of *M* sources, order 1 and 40 samples was substituted, with the MVAR coefficient matrix corresponding to the connectivity pattern for that state. This *M* × 1, 000 × 300 matrix for each condition, was the source-level data for that condition.

To fix this data in physical space, *M* randomly chosen locations just below the cortical surface on the dorsal side were selected as locations of neuronal sources, corresponding to the *M* signals in the MVAR processes. All locations were 0.8 units away from the origin of a unit sphere, where the surface of the sphere at radius 1 units represented the scalp surface. A three-shell spherical head model was used to project the data from the level of the brain to 32 standard electrode locations at the surface of the scalp. The radii of the three shells (brain, skull, scalp) were 0.88, 0.92 and 1 and their respective conductivities were 0.33, 0.0042, and 0.33. At the level of the scalp, white Gaussian measurement noise was added, whose standard deviation was (1/*SNR*) times the Root Mean Square (RMS) level of the noiseless scalp-level data, where *SNR* was signal-to-noise ratio. *SNR* had levels of 10, 3, 1, and 0.1.

Hence, the pipeline was tested under 12 different situations, i.e., every combination of 3 levels of *M* (number of sources) and 4 levels of *SNR*. To estimate sparsity level as indicated by the λ parameter, we generated independent training simulated EEG datasets for each of the 12 different parameter combinations and used GCV criterion to determine λ. Then, we generated corresponding testing datasets, with 20 datasets for each of the 12 parameter combinations. For each of these 20 datasets (for a given parameter combination), the MVAR coefficients of each state, the particular state sequence and the locations of the *M* sources were varied randomly, while all other settings were held constant. Thus, the ability of the pipeline to discriminate trials from two conditions was tested in a wide range of situations.

Since the pipeline was applied to data from both experimental conditions together, each dataset was 32 × 1, 000 × 600. These are typical dimensions of an EEG dataset combining trials from two experimental conditions. 20 such datasets were created for each of the 12 parameter combinations. For each of the 12 parameter combinations, the performance measure of model distance, averaged across the 20 datasets, was inspected to determine if the method was able to discriminate trials from experimental conditions of different time-varying network structure.

### 2.6. Experimental data

We applied the pipeline to a dataset from a P300 speller paradigm used in brain-computer interfacing (BCI) research. This comprised data from 2 participants, collected with the Wadsworth BCI2000 software from the Wadsworth Centre, New York, and is available for download online ^3^. Details of the data collection are available in Blankertz et al. (2004). Due to the high rate of intensifying rows/columns (5.7 Hz), there is a possibility of a P300 ERP from the preceding or following trials being present in the 1 s window. To preclude this, all trials in both datasets were selected on the basis that they did not have a target trial for the three preceding and five following trials. For each participant, data was analyzed from 28 electrodes covering the entire scalp, for 700 trials each from the target and non-target conditions. Each trial was from the time of stimulus presentation to 1 *s* post-stimulus. Since the sampling frequency was 240 Hz, the input dataset to the pipeline was 28 × 240 × 1400, since it was applied to trials from both conditions together. Each trial was baseline corrected by the average of activity up to 175 *ms* pre-stimulus.

## 3. Results

### 3.1. Simulation 1 - MVAR & s-MVAR comparison

**Figure 2:**
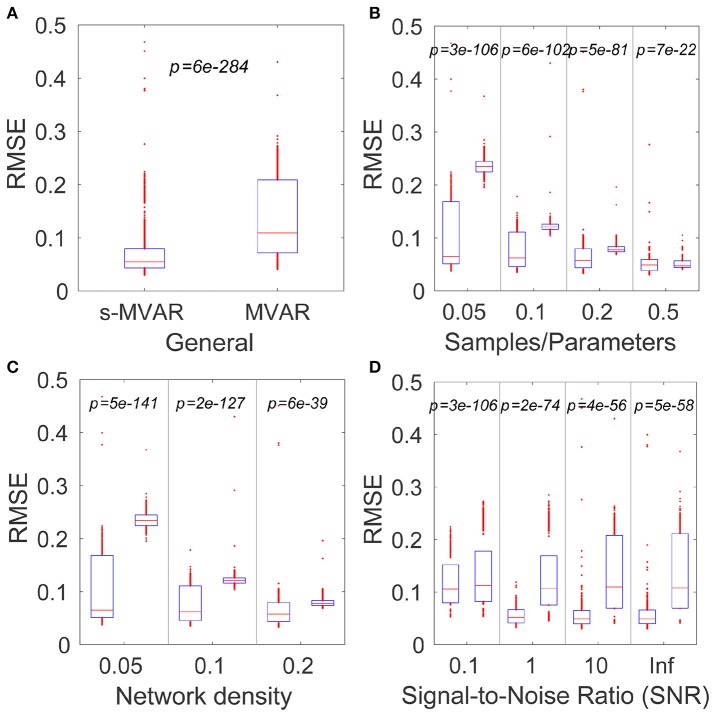
Comparing accuracies of network estimation of the MVAR and s-MVAR modeling approaches. **(A)** Box plot of estimation accuracies (RMSE) with s-MVAR (left) and MVAR (right) approaches across all factors studies. The red line indicates the median of the sample, while the lower and upper bounds of the blue rectangle indicate the 25^th^ and 75^th^ percentile levels respectively. Red dots outside blue rectangle indicate sample outliers. *p*-values are the result of one-tailed sign-tests of the hypotheses that the median of the difference between two samples is zero. Low *p*-values indicate that the accuracy of the s-MVAR estimation is higher than that of MVAR estimation. **(B)** Box plots and results of one-tailed paired sign-tests comparing s-MVAR and MVAR at different levels of the samples/parameters ratio. The s-MVAR performs better than the MVAR at all levels, especially when the ratio of samples/parameters is small. **(C)** Box plots and results of one-tailed paired sign-tests, comparing s-MVAR and MVAR at different levels of network density. s-MVAR performs better than MVAR at all levels, especially when network density is low. **(D)** Box plots and results of one-tailed paired sign-tests, comparing s-MVAR and MVAR at different levels of signal-to-noise ratio (SNR). s-MVAR performs better than MVAR at all levels. This difference is highest at high values of SNR, but most reliable at low values of SNR.

The simulations revealed that, for the factors investigated, sparse-MVAR modeling estimated the connectivity patterns more accurately than conventional MVAR modeling. The comparison across factors was found to be significant (*p* = 6*e*−284) (Figure 2A). Individual factors were also studied at each level - all comparisons were found to be statistically significant (*p* < 1*e*−20 for all).

Figure 2 illustrates each of the comparisons with box plots of the RMSE values for s-MVAR (left) and MVAR (right) respectively. The corresponding *p* values are also displayed. The *p*-values in Figure 2B indicate that the performance of the s-MVAR method is superior to MVAR at a low number of samples. Further, the *p* values in Figures 2C,D indicate that s-MVAR particularly outperforms MVAR at low network densities (high sparsity) and at low SNRs respectively. Therefore, assuming network sparsity, it is clear that s-MVAR provides more accurate estimates than conventional MVAR, in a range of situations.

To estimate the MVAR model, we used the s-MVAR model with regularization parameter λ set to 0, in order that the s-MVAR and MVAR fitting were as similar as possible. However, we also obtained the same pattern of results (not shown) when we repeated the analysis using the Vieira-Morf method (Marple, 1986) to estimate the MVAR model.

### 3.2. Simulation 2 - pipeline validation

**Figure 3:**
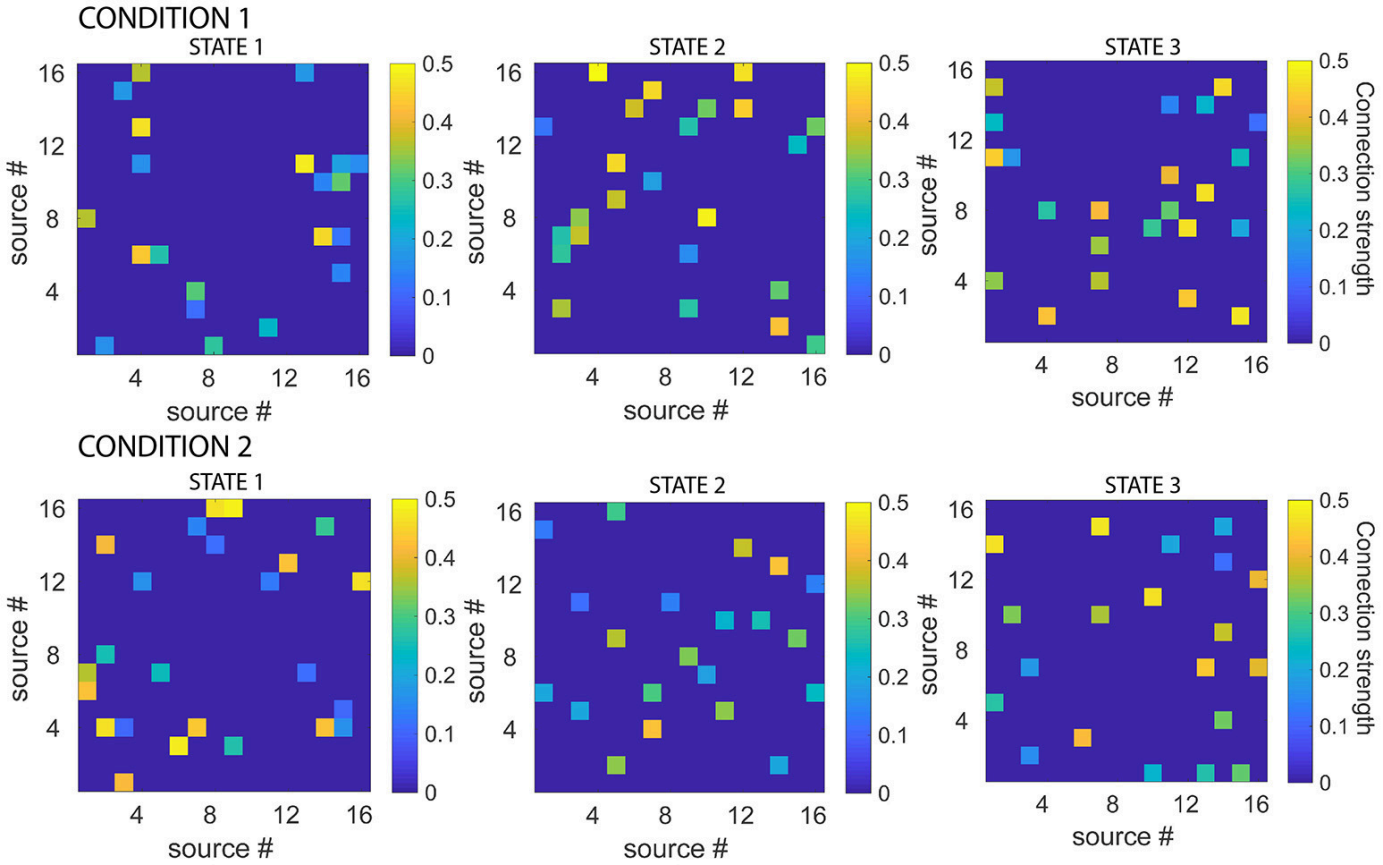
Simulated connectivity matrices for each state, for each experimental condition. While all the states had 10 % connections with strengths between 0.1 and 0.5, the actual connectivity patterns were different for each state. Note also that the functional networks defined by the states were asymmetric. Connectivity matrices are shown for example dataset, number of sources = 16, signal-to-noise ratio = 10.

To generate the simulated data, the connectivity patterns of the 6 states (2 experimental conditions, 3 states each) were first generated, by setting MVAR coefficients (lag 1) of 10% of randomly selected connections to values between 0.1 and 0.5. Given the state sequences for each experimental condition, these were used to produce electrode-level EEG data for both conditions. 20 simulated EEG datasets each, were generated for each combination of three different levels of number of sources (8,16,32) and four levels of signal-to-noise ratio (10,3,1,0.1). Figure 3 displays the connectivity patterns of the functional states of each condition, for an example dataset where the number of sources was 16 and the signal-to-noise ratio was 10.

Once a simulated dataset of 300 trials was generated, pre-processing was performed. Then, sparse-MVAR modeling was applied to non-overlapping windows of 40 *ms* each. Since the simulated data were assumed to have a sampling frequency of 1 kHz, this corresponded to windows of 40 samples. To obtain sufficient number of samples to estimate the s-MVAR model, we concatenated data from 3 consecutive trials (totally 120 samples) before performing the model estimation. After concatenation, we had 100 “trials” per condition. Since the pipeline was applied to data from both simulated conditions together, the s-MVAR modeling was effectively applied to 200 trials, with 25 windows per trial. Model order was set to 1. The λ value was calculated using the GCV criterion from 1% (50 windows) randomly selected windows of a corresponding training dataset, and the mean λ value was used. For the 12 different parameter combinations, the mean λ values obtained from the training set varied from 1.9 (number of sources=32, signal-to-noise ratio=10) to 12.8 (number of sources=8, signal-to-ratio=0.1) across the parameter combinations. Notably, the values were higher for lower numbers of sources at high signal-to-noise ratios, but these distinctions disappeared as signal-to-noise ratio decreased.

The set of vectorized connectivity matrices was then entered into *k*-means clustering with *k* set to 6, i.e., the total number of states. After clustering, each connectivity matrix could be represented by one of 6 numbers/symbols, each corresponding to a certain cluster. Results from clustering were used to produce a multi-trial sequence of symbols for each experimental condition. These multi-trial sequences were characterized using a Markov model for each experimental condition. The number of states was set to the number of clusters. Non-parametric hypothesis testing was then used to determine if the differences in the Markov model parameters of the two experimental conditions, as measured by “model distance,” are higher than expected by chance. This was done for each of the 12 parameter combinations.

We found that the pipeline could clearly discriminate trials from the two experimental conditions for each level of number of sources, down to signal-to-noise ratios of 1 (Table 1). The effectiveness of the pipeline at realistic signal-to-noise ratios suggest it is able to identify underlying functional states (by *k*-means clustering) and model the transitions between these states (by Markov modeling), thereby justifying its application to experimental EEG data.

**Table 1:** Mean (SD) of model-distance, measuring difference in model parameters for the two simulated experimental conditions, across 20 datasets.

	**SNR = 10**	**SNR = 3**	**SNR = 1**	**SNR = 0.1**
Number of sources = 8	–68.2 (82.8)	–47.8 (143)	–6 (4.9)	–1.2 (0.3)
	*p* < 0.001	*p* < 0.05	*p* < 0.05	*p* > 0.05
Number of sources = 16	–252.1 (302.2)	–73.6 (162.8)	–24.3 (48.2)	–1.3 (0.2)
	*p* < 0.001	*p* < 0.001	*p* < 0.001	*p* > 0.05
Number of sources = 32	–1.8263*e*+03 (843.3)	–179.1 (196.2)	–28.4 (29.7)	–1.2 (0.3)
	*p* < 0.001	*p* < 0.001	*p* < 0.001	*p* > 0.05

*Average p-values across 20 datasets, obtained from non-parametric hypothesis testing are also shown. The p-values indicate that trials from the two experimental conditions can be discriminated at levels of numbers of sources, down to signal-to-noise ratio = 1*.

On one example trial (number of sources=16, signal-to-noise ratio = 10), we also tested the ability of the pipeline to discriminate conditions when trials were randomly jittered in time. To do this, we jittered each trial (before concatenation) by randomly specified numbers of samples between 1 and 20 (i.e., 1–20 ms). Notably, the pipeline was able to discriminate between the two experimental conditions (*p* < 0.001) even in such a scenario.

**Figure 4:**
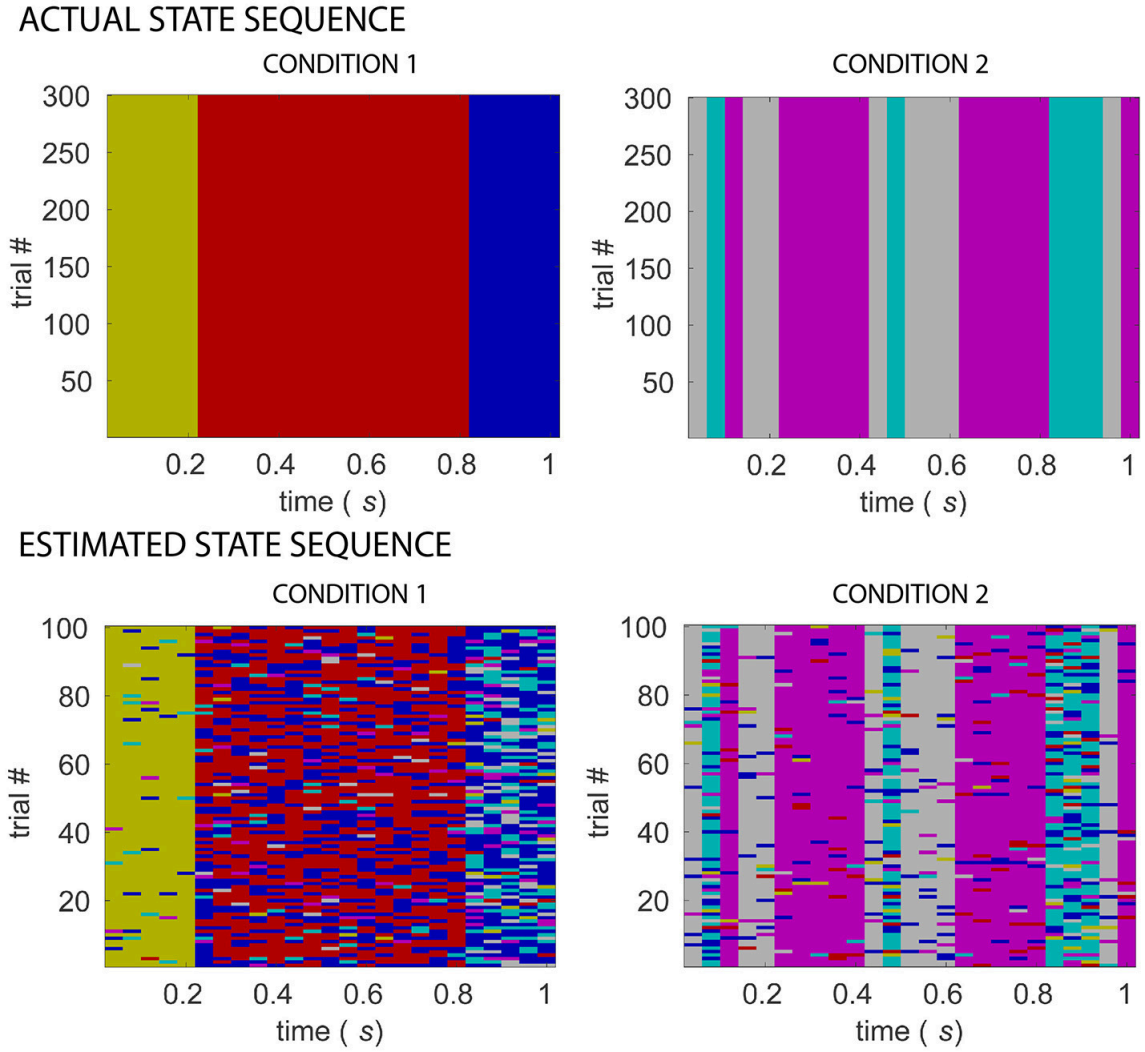
Comparing actual and estimated sequence of states for two simulated experimental conditions. Matrices in the top row display multi-trial state sequences for simulated conditions 1 and 2, different colors indicating different states. Matrices in the bottom row display state sequences inferred from 32-electrode EEG simulated data of the two conditions, following s-MVAR and clustering stages, for an example dataset for which number of sources = 16, signal-to-noise ratio=10. A rough correspondence is evident between assumed and estimated state sequences, for both experimental conditions. There are however a number of mis-allocated states, which might be due to blurring due to volume conduction, of the differences between the underlying source-level functional networks.

**Figure 5:**
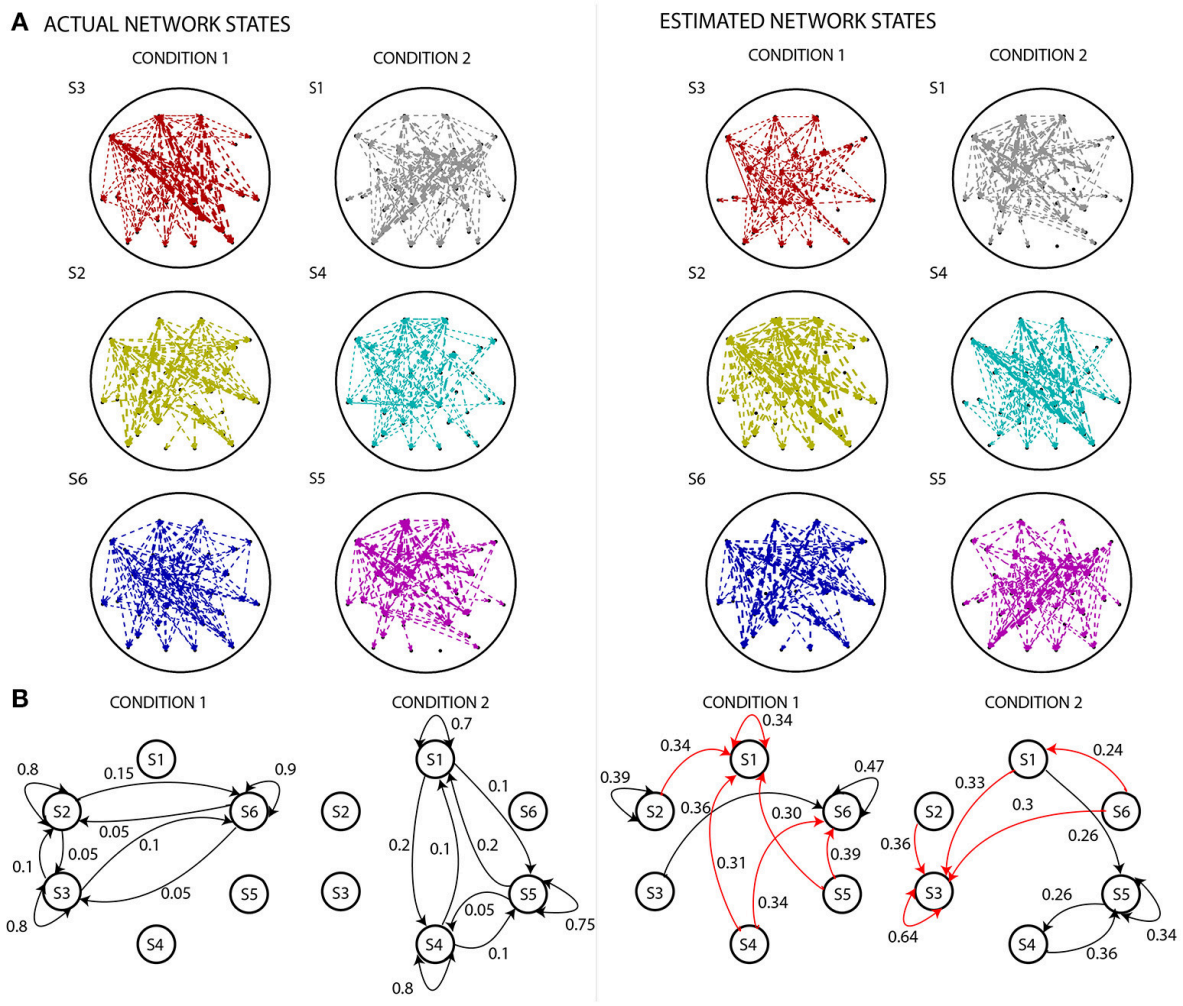
Comparing actual states and state diagrams with estimated states and state diagrams. **(A)** The left panel displays the EEG electrode-level connectivity patterns of the states for the two experimental conditions. The right panel displays the corresponding electrode-level connectivity patterns of the states, after applying the analysis pipeline. The width of the lines are linearly proportional to the value of s-MVAR coefficients. Apart from states S2 (dark yellow) and S6 (blue), there are substantial differences between the estimated and actual electrode-level connectivity patterns. For both left and right panels, the electrode-level connectivity patterns for a state are obtained by averaging functional network representations (i.e., the inputs to the clustering stage) belonging to that state. Note, only connections within the top 10^th^ percentile strength are displayed. **(B)** The left panel displays state diagrams of both simulated conditions, i.e., probabilities of transition from each state to all states including itself. The right panel displays transition probabilities inferred by estimating the Markov model for each of the conditions. While some of the transition paths are correctly revealed (black lines), many spurious paths are also produced (red lines). Only values in top 25 percentile are shown.

Figure 4 shows a comparison of the actual and estimated sequence of states for two simulated experimental conditions, for the same example dataset as in Figure 3 for which number of sources=16, signal-to-noise ratio=10. The estimated sequence of states can be obtained from the pipeline after the s-MVAR and clustering stages are performed. While there is a rough correspondence between the actual and estimated sequence of states, there are also a number of state mis-allocations. These mis-allocations might be caused by linear mixing due to volume conduction. Once the Markov modeling stage is performed, characteristic electrode-level functional networks for each state and probabilities of transition between states can be obtained. Figure 5 shows actual and estimated electrode-level functional network for each state, as well as actual and estimated transition probabilities between states for the same example dataset. Apart from states S2 (dark yellow) and S6 (blue), the mis-allocation of states in the clustering stage, presumably due to volume conduction, produces distortion of the characteristic functional networks of the states, as compared to the actual pattern of connections (Figure 5A). The mis-allocation of states in the clustering stage also produces distortions in the estimated probabilities of transition between states (Figure 5B, spurious paths shown in red). Thus, while the pipeline is able to discriminate between trials of the two experimental conditions, simulations suggest that estimated electrode-level connections patterns of each state, and estimated probabilities of transitions between states are distorted versions of the ‘ground-truth’.

### 3.3. Experimental data

As mentioned above, the pipeline was also applied to 700 target and 700 non-target trials from a BCI oddball paradigm. The sampling frequency was 240 Hz. To obtain sufficient number of samples (120 samples) to estimate the s-MVAR model at the required window width of 40-50 *ms*, we could have concatenated 12 samples (50 ms) of data from 10 consecutive trials or 10 samples (40 ms) of data from 12 consecutive trials. We chose the former since it makes weaker assumptions about stationarity of the s-MVAR process across trials. Hence, we applied the pipeline simultaneously to 70 ‘trials’ from the target and non-target conditions each. The number of non-overlapping windows for each ‘trial’ was 20.

**Figure 6:**
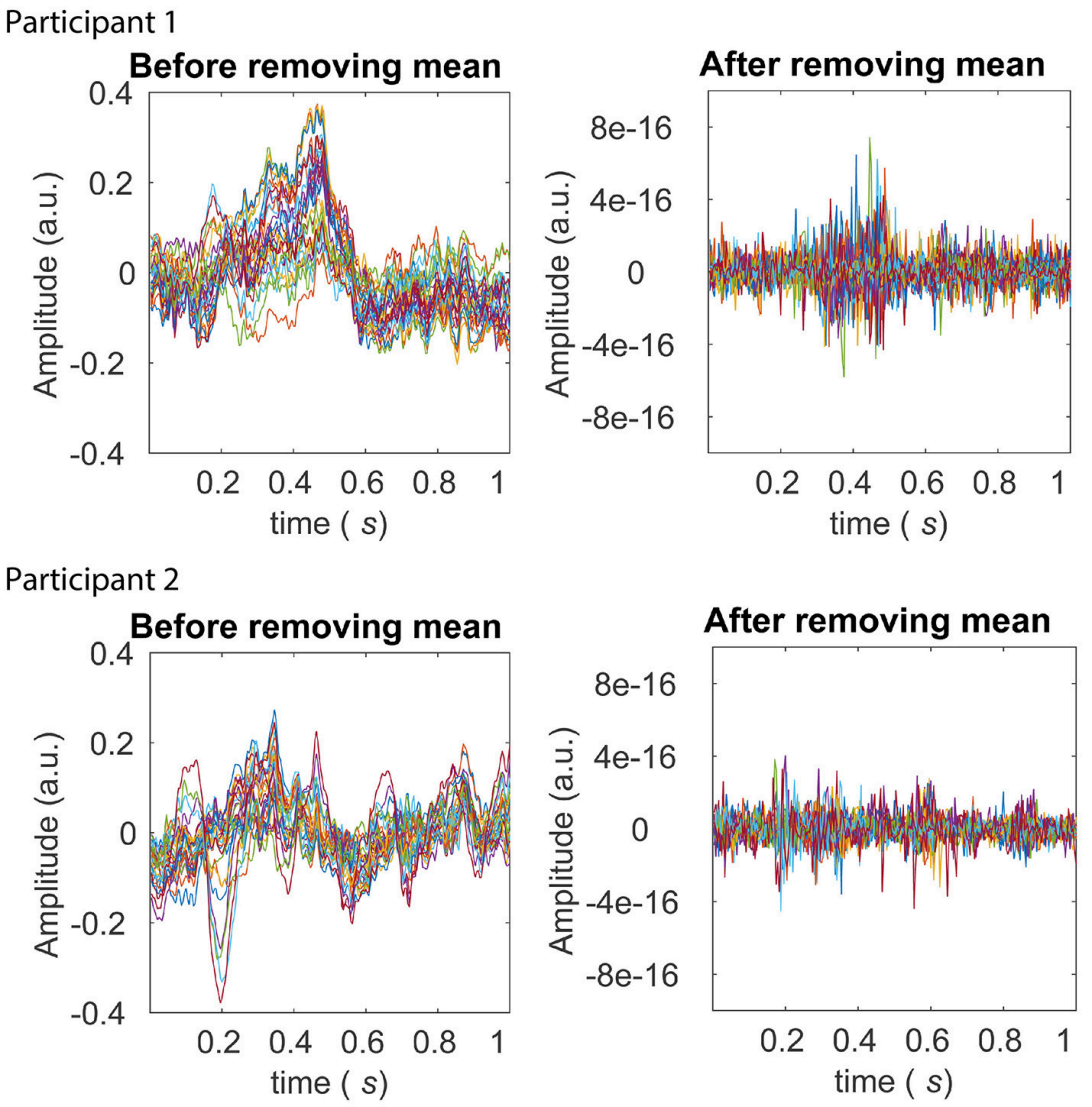
Averages across trials before and after the pre-processing stage of *z*-scoring across trials (within each experimental condition). The left panels in the top and bottom row display averages across trials after *z*-scoring across samples for Participants 1 and 2 from the BCI dataset, respectively. Averages from each of the 28 electrodes are shown in different colors. The P300 ERP is evident between around 0.2 and 0.5 seconds for both participants, in the left panels. The right panels in the top and bottom row display averages across trials for Participant 1 and 2 respectively, after the further pre-processing stage of *z*-scoring across trials. It is clear from scale of the right panels that across electrodes, any first-order (mean) sources of non-stationarity are eliminated.

First, we performed the pre-processing, i.e., *z*-scoring across samples and then across trials. This eliminated first-order (mean) and second-order (standard deviation) sources of non-stationarity from the dataset. Note that removing the mean across trials effectively removes ERPs from the data. Figure 6 displays the average across trials, for each of the channels, before and after the *z*-scoring across trials was performed. The scale of the plots on the right compared to those on the left clearly reveal that first-order sources of non-stationarity are eliminated.

After pre-processing, we performed the sparse-MVAR modeling on each non-overlapping window. The model order for this was determined as 1 for both participants, by estimating the modal value of the model order on 10% of randomly selected windows for each participant separately. The sparsity level parameter λ, was also estimated as the mean value obtained from estimating it on 10% of randomly selected windows, using the GCV index. The λ value for Participant 1 was 1.03 and was 0.89 for Participant 2.

We then ran the clustering stage with the number of clusters *k*, set from 2 to 8 (intervals of 1). For each of these values of *k*, we obtained a multi-trial state sequence and corresponding Markov model parameters for each experimental condition, which were then used to discriminate trials between conditions based on the measure of 'model distance'. Please see Table 2 for results.

**Table 2:** Model Distance (MD) and corresponding *p*-values for two participants (Participant1 and Participant 2), for a range of values of *k*, i.e., the number of clusters.

		***k* = 2**	***k* = 3**	***k* = 4**	***k* = 5**	***k* = 6**	***k* = 7**	***k* = 8**
P1	MD	–0.4	–0.63	–2.78	–2.58	–3.75	–3.91	–4.91
	*p*-value	>0.05	>0.05	<0.05	>0.05	<0.05	>0.05	>0.05
P2	MD	–0.79	–1.42	–1.95	–2.49	–3.7	–4.8	–5.5
	*p*-value	>0.05	>0.05	= 0.05	= 0.05	<0.01	<0.05	>0.05

*p-values are indicated in brackets. Both participants had statistically significant model distances at k=4 and k=6 (p-values ≤ 0.05), while Participant 2 also had statistically significant model distance at k=5 and 7 (p-values ≤ 0.05)*.

For both participants, the estimated models from the two conditions were found to be different at *k* values of 4 and 6 (*p* ≤ 0.05). Participant 2 also had statistically significant model distance for *k*=5 and 7 (*p* ≤ 0.05). The ability of the pipeline to discriminate trials from two experimental conditions suggest that it is able to identify functionally relevant states (by *k*-means clustering) and model the transitions between these states (by Markov modeling). We also inspected the time courses of example s-MVAR processes, whose parameters had been estimated from the EEG data of Participant 1 and Participant 2. The resemblance of these processes to typical EEG dynamics is also consistent with functional relevance of the states they represent (Figure S2).

**Figure 7:**
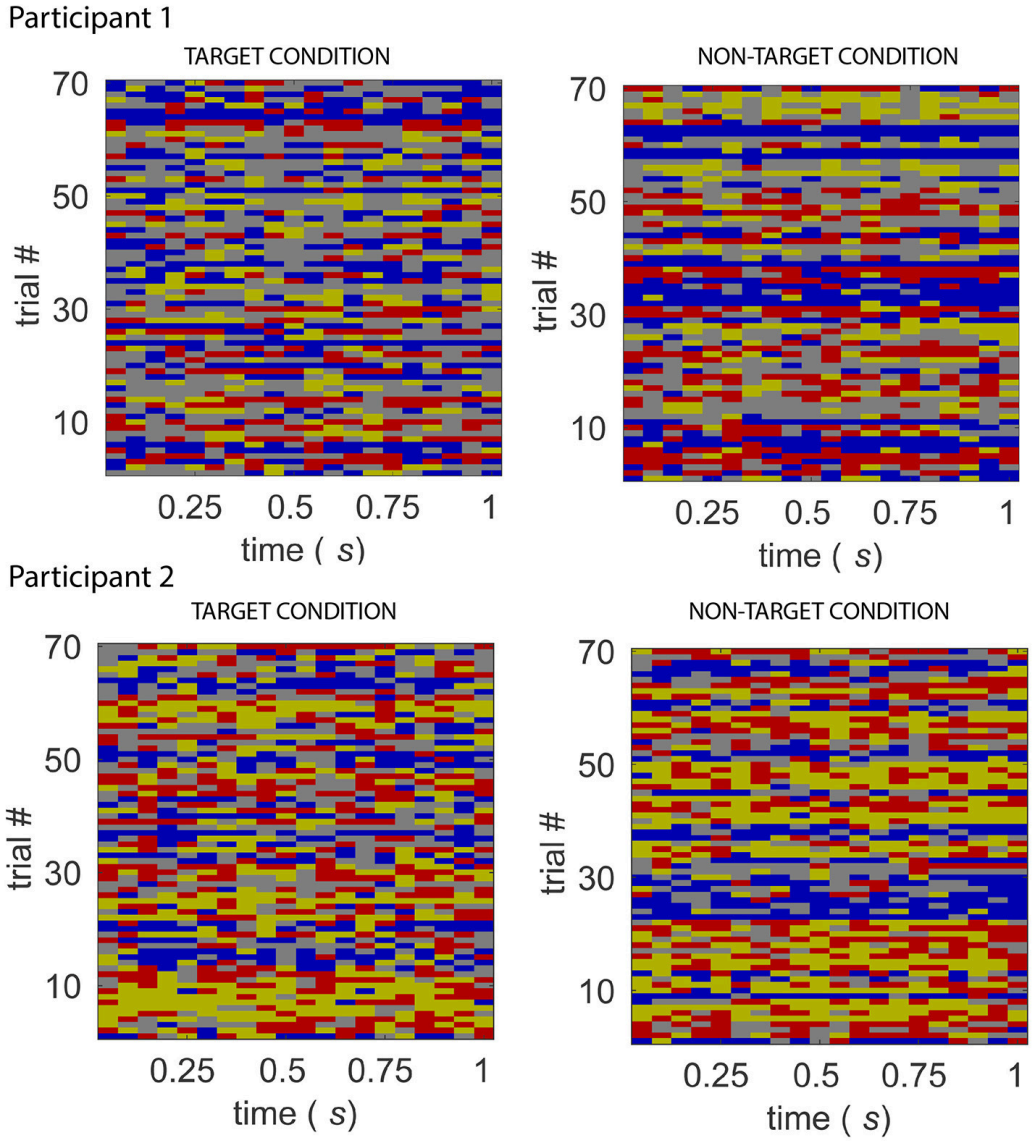
Sequence of states for the target and non-target conditions, from Participant 1 **(top)** and 2 **(bottom)** of the BCI dataset. The sequence of states for 70 “trials” of target and non-target conditions. A feature of the state sequences across participants and experimental conditions is the high probability of the system staying in the same state.

**Figure 8:**
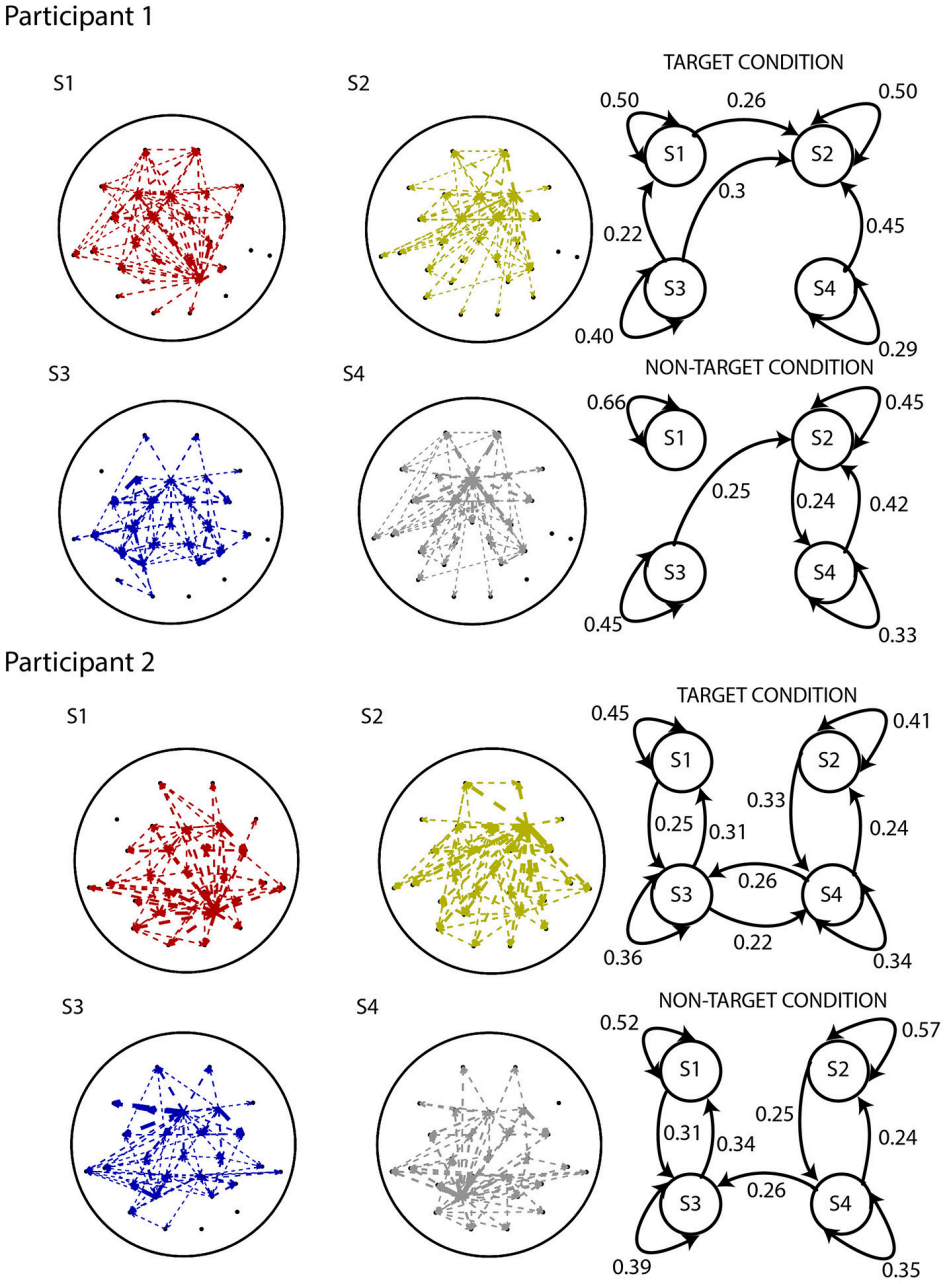
Results from applying the analysis pipeline to Participants 1 **(top)** and 2 **(bottom)** of the BCI dataset. EEG electrode-level connectivity patterns **(left)** for four states, obtained by averaging the functional network representations belonging to that state. The width of the lines are linearly proportional to the values of the s-MVAR coefficients.Electrode-level connectivity patterns of states S1 (red), S2 (dark yellow) and S3 (blue) are highly similar between Participants 1 and 2. State diagrams **(right)** displaying transition probabilities for both target and non-target conditions. Only the transition probabilities above 0.2 are shown. Due to distortion of the electrode-level network and spurious transition paths that can occur, as demonstrated by our simulations, interpreting these networks and state diagrams in terms of underlying neuronal processes is difficult.

Figure 7 displays the multi-trial sequence of states for Participants 1 and 2, when *k* was set to 4. For both participants, the probability of staying in the same state is high for each of the states, for both target and non-target experimental conditions. This might suggest that the temporal resolution obtained from identifying states on short non-overlapping windows is sufficient to detect state changes. Figure 8 displays the characteristic electrode-level functional networks of each state for each of the participants, as well as their corresponding state diagram (indicating probability of moving between states). Remarkably, Participants 1 and 2 have highly similar electrode-level connectivity patterns for S1 (red), S2 (dark yellow) and S3 (blue). The similarity of electrode-level connectivity patterns between Participants 1 and 2 suggests that the functional states they represent might be neuro-physiologically relevant.

The state diagrams for Participant 1 suggest that there is a stronger tendency to switch between states in the target than the non-target condition, while no such difference is apparent in Participant 2. However, interpretation of the electrode-level connectivity patterns or state diagrams in terms of underlying neuronal processes is difficult, due to distortion of networks and spurious transition paths that manifest, as demonstrated in our previous simulations.

An implication of Markovian dynamics is that the distribution of state dwell times follows a geometric distribution. Inspection of the histogram of dwell times for each participant, for each experimental condition, does indeed reveal that the distributions of dwell times of each of the states approximates the geometric case (please see Figure S3).

## 4. Discussion

### 4.1. Significance

In this work, we proposed and applied a pipeline to characterize the time-varying structure of functional networks in a cognitive neuroscience task context. Using scalp-level simulated data generated with a realistic EEG forward model, we were able to demonstrate that trials from two experimental conditions could be discriminated under a range of simulated scenarios. This justified the application of the pipeline to experimental data. Applying the pipeline to experimental data of two participants performing a BCI oddball task, we reported statistically significant discrimination between trials from the two experimental conditions, using only the Markov model parameters. That we were able to achieve this on such a parsimonious representation of the EEG activity, characterized primarily by the transition probabilities between states, is of relevance. Notably, there was similarity between the two participants, in the electrode-level connectivity patterns of the functional states. Both the ability to discriminate trials between conditions on simulated data and experimental data, and the between-participant similarity in the connectivity patterns of the identified functional states suggest that the pipeline is able to identify the underlying functional states (by *k*-means clustering) and transitions between these states (by Markov modeling).

The pipeline can thus be used to compare two experimental conditions, specifically in terms of their patterns of fast transitions between identified functional states. To our knowledge, this is the first instance of the use of the Markov modeling framework to analyse time-varying networks in EEG/MEG data. sparse-MVAR modeling has been used to estimate functional networks in *f* MRI (Valdés-Sosa et al., 2005) as well as EEG/MEG (Haufe et al., 2009), but to our knowledge, this is also the first time its potential to estimate networks from a small number of samples is being harnessed to infer time-varying networks in EEG/MEG data. The information provided by the pipeline is orthogonal and hence complementary to other analysis approaches. Specifically, the first pre-processing stage of *z*-scoring across channels removes spatial differences in amplitudes of channels that are used in EEG microstate analyses, while the second pre-processing stage of *z*-scoring across trials removes the conventional ERP average.

### 4.2. Relevance to cognitive neuroscience

In the context of cognitive neuroscience, tracking changes in the pattern of the functional network as a task is being performed, is particularly relevant to inform models of the neuronal processes underlying cognition. EEG/MEG are well suited to capture these network changes, due to their millisecond-level temporal resolution and direct recording of electrophysiological activity. Using analysis methods which capture these changes, like the method we propose, allow researchers to dispose off the limiting assumption of static connectivity, and test detailed hypotheses of rapid changes in interaction patterns between brain regions, as a task is being performed.

There is also a growing literature on emulating MEG/*f* MRI activity using whole-brain computational models of brain function (see for e.g., Cabral et al., 2014; Nakagawa et al., 2014). Many of these modeling efforts are constrained to using a static connectivity framework to compare model-generated data with experimental data. Through the use of the proposed method, the model's ability to generate non-stationary network dynamics can be evaluated, and results from applying the pipeline offers novel features (e.g., transition probabilities) to compare model-generated data with experimental data, as well as to tune free parameters of the model.

### 4.3. Comparison with other methods

Our approach bears similarities to methods proposed in Wilke et al. (2008), Sommerlade et al. (2009), and Hu et al. (2012). While these approaches combined an MVAR model with a Kalman-filter/smoothing model, we combined a sparse MVAR model with a Markov model via an intermediate clustering stage to reduce the dimensionality and complexity of input to the Markov model. These previous approaches are powerful in that they furnish a sample-by-sample estimate of the functional network, in contrast to our approach of estimating the network on non-overlapping windows. However, we considered that the assumption of the Markov model, with regard to the discrete nature of state space, to be appropriate. Related to this, we considered that a Markov model, with its parameterisation of transitions between states, is better suited to describe observed metastable/multistable network dynamics. As mentioned, this metastable/multistable perspective has also been adopted by earlier approaches and is supported by empirical data (Koenig et al., 2002; Ito et al., 2007; Baker et al., 2014).

A method which is close to ours in terms of modeling assumptions is the HMM-based method proposed in Baker et al. (2014). The method furnishes a sample-by-sample estimate of HMM parameters and since the HMM is fit to source-reconstructed MEG data, the states can be related to patterns of activity in the brain itself, rather than at the electrode/sensor level. However, unlike the method proposed here, the states do not correspond to functional networks but rather multivariate patterns of activity. Another method similar to ours, that has been proposed, is that by Hirayama and Ogawa (2014). In this paper, the authors introduce a unified method which estimates source-level activity via Blind Source Separation (BSS) and infers latent co-activity patterns corresponding to 'putative' states via a mixture model. Due to the simultaneous estimation of both stages, the model is estimated more accurately than if the parameters were estimated in two separate stages (Hirayama and Ogawa, 2014). Apart from details of the mixing matrix, the estimated model furnishes details of the co-activation patterns and state probabilities. As in the Baker et al. (2014) paper, the definition of a state is more general than a pattern of functional connectivity. Furthermore, while individual states are identified, the probabilities of transitions between these states are not parameterized in the model.

Two related methods which are similar to ours are the ones proposed in Daly et al. (2012) and Vidaurre et al. (2016). In Daly et al. (2012), patterns of phase-locking at the electrode level were described using complex network measures (mean clustering coefficient) which were in turn used as observables to an HMM. In Vidaurre et al. (2016), the observables to the HMM were parameters from the MVAR model of MEG source-reconstructed time series. A key difference between our method and these is that we use a Markov model rather than a Hidden Markov Model (HMM), to characterize time-varying network dynamics. This produces a more parsimonious representation than the above methods, of the EEG activity patterns indexing a given cognitive process, based primarily on the matrix of transition probabilities between states. Over and above the parsimony of the representation, we demonstrated the sufficiency of the representation to discriminate trials from two conditions. The representations of the functional networks also follow the principle of parsimony, through our use of the s-MVAR rather the conventional MVAR modeling framework.

### 4.4. Limitations

The primary limitation of the pipeline in its current form is interpretability of its results, particularly how they relate to source-level networks underlying each functional state. This limitation has two aspects. One is that in its current form, the pipeline yields connectivity patterns underlying states at the level of *electrodes* rather than at the level of brain regions. This problem could be addressed by including a stage for reconstructing source-level activity through an inverse projection, for e.g., Minimum Norm Estimation Hauk (2004) or Beamforming van Veen et al. (1997), before the s-MVAR estimation stage. Assuming a fine parcellation of brain regions however, this would dramatically increase the number of parameters to be estimated in the s-MVAR model and potentially increase the window width - the ability to track rapid changes in functional networks would be diminished. An alternative approach would be to project the estimated s-MVAR coefficients to obtain directed connectivity patterns at the level of brain regions (Michalareas et al., 2013). Compared to first projecting the EEG/MEG data to the source-level and inferring the MVAR model from the source time-series, this procedure would greatly reduce the number of MVAR coefficients to be estimated and likely lead to more accurate estimation. Importantly, it would also circumvent problems of sign ambiguity of source-reconstructed time-series, as encountered for e.g., in Vidaurre et al. (2016).

The second aspect limitation is that, as demonstrated in the simulations, the electrode-level connectivity patterns of the states are themselves distorted versions of the ground-truth electrode-level connectivity patterns, as also are the estimates of transition probabilities between states. This is likely because of mis-allocation of states during the clustering stage, due to errors in the s-MVAR coefficients. These errors might be caused by linear mixing due to volume conduction. Indeed, we were able to estimate the clusters with perfect accuracy when we applied the pipeline to source-level simulated data (not shown). It is known that the linear mixing due to volume conduction distorts estimates of functional networks at the level of electrodes Brunner et al. (2016) as well as at the level of brain regions (Hillebrand et al., 2012). A potential solution to this could be to extend the present model to account for the linear mixing due to volume conduction, that occurs as activity travels from the brain to the scalp. A further limitation of the pipeline is that similar to MVAR, the s-MVAR framework combines time-delayed dependencies in both signal amplitude and phase, but these are likely to be functionally independent phenomena(Bastos and Schoffelen, 2016).

Due to the short time windows we use, we use model order of 1 to estimate the sparse-MVAR models. Since the model order is twice the maximum number of peaks in the frequency spectrum between a given pair of EEG channels (Florian and Pfurtscheller, 1995; Schlogl and Supp, 2006), the frequency resolution of the sparse-MVAR coefficients in our fitted models is poor. Hence, while the pipeline is able to detect time-lagged correlations between oscillatory activities of brain regions, the low model order does not allow us to determine the frequency at which these correlations are present. A further limitation of the method is that the *k*-means procedure can produce different results for different runs, unless the initial cluster centroid positions are made identical across runs. Alternatively, deterministic clustering procedures, for e.g., some forms of spectral clustering (von Luxburg, 2007) could be employed.

### 4.5. Caveats

It is also useful to consider possible confounds to interpreting the results in terms of time-varying connectivity. While first-order (mean) and second-order (standard deviation) sources of non-stationarity were effectively removed through pre-processing, network-level sources of non-stationarity might be present in the data, for e.g., the variation of zero-lag correlation in channels over time. Further, one should be cautious in making inferences about the nature of state changes due to the apparent validity of an Markov model approach, i.e., whether deterministic or stochastic. While the Markov model is a stochastic model, it is agnostic with regard to whether the sequence of states is deterministic or stochastic, provided the model parameters are stationary across trials. In this regard, the framework is general enough to characterize an identical/deterministic sequence of states since this would be a special case of the model.

### 4.6. Future work

Notably, the use of the Markov model framework lays a foundation for useful extensions of the method, particularly to improve neuro-physiological relevance of the results. Importantly, the estimation of the s-MVAR model and Markov model could be combined, allowing for greater temporal and spectral resolution. As mentioned above, once the MVAR coefficients corresponding to each state have been estimated at the electrode level, it has been demonstrated in Michalareas et al. (2013) that it is possible to obtain source-level MVAR coefficients by combining the original coefficients with the lead-field and inverse operators (Michalareas et al., 2013). Hence, source-level functional networks corresponding to each state can be obtained without compromising the ability to resolve state changes at a fine temporal scale. An alternative would be to extend the present model to account for the linear mixing due to volume conduction. This would also enable a more direct interpretation of the model parameters in terms of underlying neurophysiology. Work by Haufe et al. (2009) represents work in this direction and could be built upon.

Notably, the pipeline is only applicable to single-subject EEG/MEG data. To extend it to be applicable at the group-level, the *k*-means procedure could be applied to the s-MVAR coefficients (from each window, each trial, each condition), *of all subjects*. This would impose a common labeling and number of functional states across the different subjects. The Markov model parameters could then be estimated from each subject, each experimental condition separately, and the corresponding sets of transition probabilities could be compared between experimental conditions. This is also a topic for future work.

## 5. Conclusions

In this paper, we introduce a pipeline to analyse time-varying networks in EEG task-related data. The purpose of the method is to enable cognitive neuroscientists to test detailed hypotheses about fast changes in functional interaction patterns as a task is being performed. We established validity of the pipeline by demonstrating its ability to distinguish between trials from two simulated experimental conditions with different time-varying network structure, in a range of simulated scenarios. On applying the method to BCI oddball data from two participants, we obtained statistically significant discrimination between trials from target and non-target conditions. Apart from demonstrating the method's ability to discriminate between conditions using just the Markov model parameters, this work also marks the first application of the Markov model framework to infer time-varying networks from EEG/MEG data. Thus, the method represents a promising direction in the effort to elucidate time-varying networks from EEG/MEG data.

## Author contributions

NW, ID, and SN contributed to conception and design of the method. NW performed the analyses and programming. NW wrote the first draft of the manuscript. ID and SN contributed substantially to revision of the manuscript. All authors read and approved the submitted version.

### Conflict of interest statement

The authors declare that the research was conducted in the absence of any commercial or financial relationships that could be construed as a potential conflict of interest.

## Footnotes

^1^https://www.cs.ubc.ca/~murphyk/Software/HMM/hmm.html

^2^https://github.com/nitinwilliams/eeg_meg_analysis/tree/master/sMVAR_MM_toolbox

^3^http://www.bbci.de/competition/iii/
