# Supplementary material for: Markov Model-Based Method to Analyse Time-Varying Networks in EEG Task-Related Data
Source: Front Comput Neurosci. 2018 Sep 21;12:76. doi: 10.3389/fncom.2018.00076 (PMC6160873; doi:10.3389/fncom.2018.00076)
Supplement: Supplementary file 1 [file Presentation_1.PDF]

Supplementary Material:
Markov Model-based method to analyse time-varying networks in EEG task-related data

N. J. Williams, I. Daly and S. J. Nasuto
*Correspondence:
N. J. Williams
nitinwilliams@gmail.com

1 SUPPLEMENTARY FIGURES

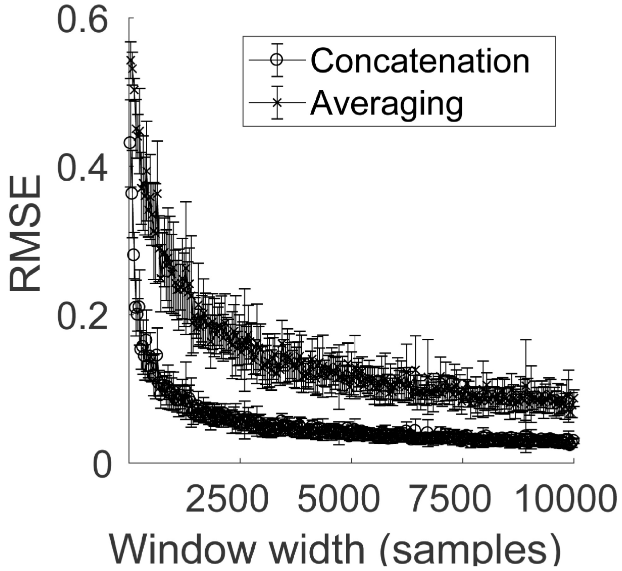

Figure S1. Comparing approaches to combining data from multiple trials in estimating s-MVAR model. To determine a suitable method to combine data from multiple trials, we compared: 1.) Concatenating data from multiple trials, after removing mean across samples from each individual trial, and 2.) Computing individual regression terms of the model solution for each individual trial and then averaging them for use by the Majorisation-Minorisation algorithm for iterative refinement. To compare the two approaches, we estimated s-MVAR models for windows of lengths between 40 samples and 10,000 samples (intervals of 40), combining 3 trials each time. The number of channels was set to 20 and the model order was set to 1, so a 20×20 MVAR coefficient matrix was generated by randomly choosing values between 0 and 1. This matrix was multipled by a matrix of input signals X, with values drawn from a Gaussian distribution with mean 0 and standard deviation 1. The output signal Y , was then obtained by adding Gaussian noise to the resulting matrix, again drawn from a distribution with mean 0 and standard deviation 1 across samples. The s-MVAR model was estimated from this dataset via the two approaches, with RMSE used as a measure of performance (lower values indicating better performance). Ten replications were performed and the mean RMSE value across replications was taken to indicate the respective performances of the two ways of combining trials for s-MVAR estimation. The results are plotted in the figure, with markers indicating the sample mean and error bars indicating 1 standard deviation above and below the mean. The results reveal that for each level of window width studied, s-MVAR modeling by concatenating trials produces more accurate estimates than by averaging the regression terms.

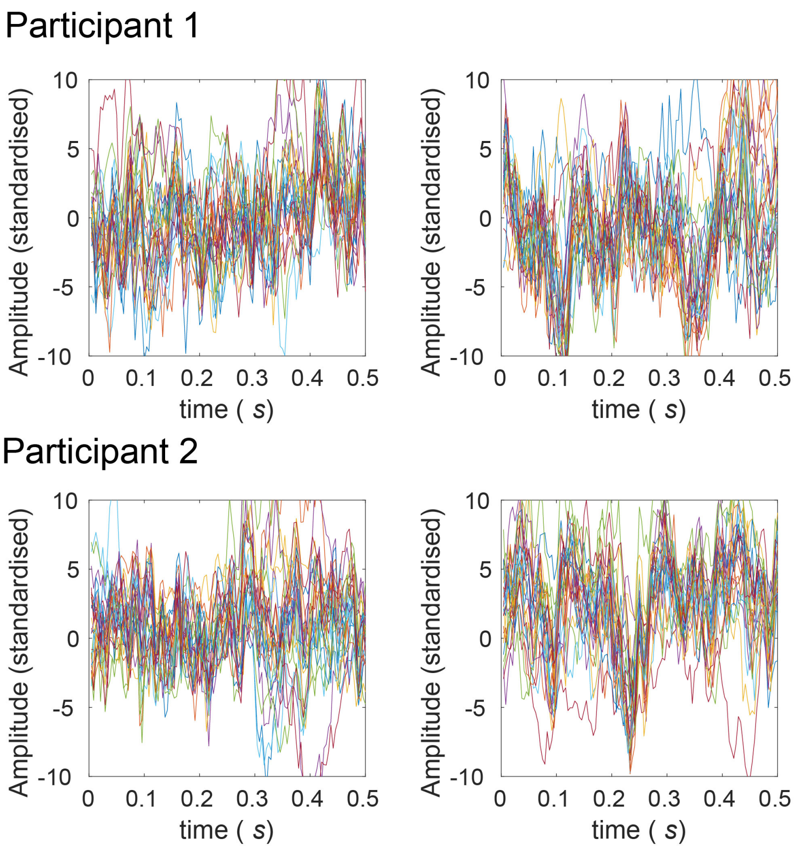

Figure S2. Examples of s-MVAR processes, with parameters estimated from EEG data of Participant 1 and Participant 2 in BCI oddball task. Dynamics of example s-MVAR processes from both Participant 1 (top row) and Participant 2 (bottom row) resemble typical EEG dynamics.

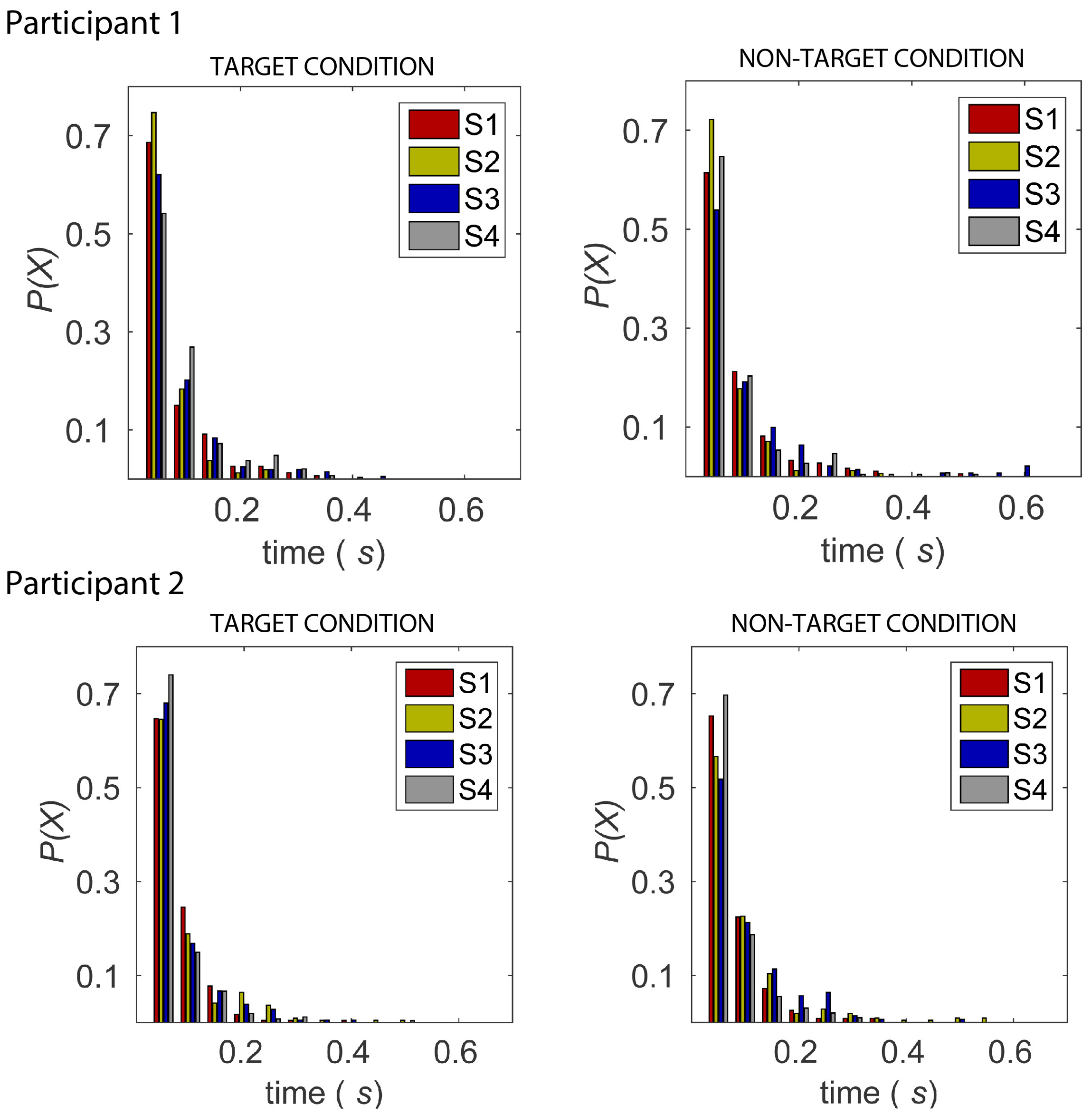

Figure S3. Histograms of dwell times for the four states, for target and non-target conditions, Participants 1 and 2. Across participants and experimental conditions, the histograms for each state approximate a geometric distribution, suggesting that the assumption of Markovian dynamics is valid.
